# The Modern Drama: A Contribution to Mental Dietetics
†*The Eighth Commandment*. By Charles Reade. London: Trübner and Co. 1860.


**Published:** 1860-10-01

**Authors:** 


### Art. IV.—THE MODERN DRAMA:

### A CONTRIBUTION TO MENTAL DIETETICS.†

If a new Jeremy Collier were now to arise, his picture of the "profaneness and immorality" of the English stage would necessarily be much more subdued in tone than that which aroused so much anger and indignation among our poets and players at the close of the seventeenth century. He could not charge our theatre with gross immodesty and indecency, or bring forward the works of Terence, Plautus, Seneca, Æschylus, Sophocles, and Euripides, to prove that the Pagan Greeks and Romans displayed greater purity in their writings than Christian Englishmen. He could not charge our dramatists with a fondness for profanity, or with recognising cursing and swearing, coupled with abuse of religion, as the distinguishing evidences of wit and good breeding. He could not accuse them of systematic and scurrilous ridicule of the

† *The Eighth Commandment.* By Charles Reade. London: Trübner and Co. 1860.

clergy, or find any play of modern date in which a minister of religion is spoken of, like Dominick in *The Spanish Friar*, as "a parcel of holy guts and garbage," with "room in his belly for his church steeple." He could not allege that, in the dramatic works of the present day, all the principal characters are represented as worthless and vicious, and yet receive no punishment at the end; and if, in support of his censures against the stage, he cited the high authority of Theophilus Antiochenus, Tertullian, Clemens Alexandrinus, Minutius Foelix, St. Cyprian, St. Chrysostom, St. Hierom, or St. Augustine, readers would be apt to smile at these hallowed names, and to question the propriety of bringing forward the Fathers of the Church as authorities upon the subject of the English theatre and the English drama of our own time.

Has our stage grown so pure, then, it may be asked, in this the middle of the nineteenth century, that it needs no castigation at the hands of a modern censor? Have we no Drydens, no Congreves, no D'Urfeys, giving vice a modish, agreeable air, rendering ribaldry fashionable, impure actions patterns for imitation, and filthy witticisms standards of polite conversation? In our dramatic literature are there no reproductions of *Love for Love, The Mock Astrologer, The Provoked Wife,* or *The Old Bachelor?* Have we quite cleared out the Augean stables and kept them so carefully attended to ever since, that no fresh impurities have been allowed to accumulate there?

Undoubtedly, the English stage of the present day is free from all the more glaring and obvious vices which prevailed when the great nonjuring divine wrote his famous essay. We do not now habitually make light of the marriage tie in our theatre. Adultery is not now systematically elevated into a virtue it behoves all dashing fellows of spirit to practise. If we wish to gain favour for the fallen we take good care to throw around them, first of all, a veil of sentiment and pathos. Our poets do not now put indecent verses into the mouths of women and young girls, or indulge in equivocations and inuendo worthy only of prostitution and the stews. The gallants of our stage do not, like the wits and fine gentlemen described by Macaulay, unceasingly utter ribaldry of which a porter would be ashamed, or call upon their Maker every five minutes "to curse them, sink them, confound them, blast them, and damn them."

No! our theatre has at least learnt good manners since the glorious days of the Restoration, and is no longer a rendezvous of profligacy or a recruiting ground for the brothel and the bear-garden.

Yet we think it would not be difficult to show that the stage is still far from reaching the high intellectual standard it ought to attain; that it by no means exercises all the ethical or æsthetical influence it ought to exercise; and that its shortcomings, innocent as they may be when compared with its former vices, reflect no great credit upon our literary reputation as a nation. We had almost said that they cast a distinctive reproach upon it.

What should be the object of dramatic entertainment? Are plays merely meant to gratify the senses, to please the eye with dazzling colours, to divert the ear with amusing dialogues, to tickle the fancy with ingenious stage combinations? Many, doubtless, will be ready to reply with an unhesitating affirmative to these questions. They will say—"We go to the theatre to be amused, not to be sermonized; we go there as a relief from the cares, the occupation, and the anxieties of daily life: if we want to gain instruction, we can apply for it in our books; and if our consciences ever grow uneasy, the pulpit will restore them to calmness. Let the theatre, therefore, be dedicated to our amusement, and to our amusement only."

Now we are not writing in the interests of cant, still less with a view of slyly insinuating ourselves into the good graces of Exeter Hall; let us, therefore, admit that the theatre *is* a place of recreation, that it *is* a place where we go to seek distraction amid the troubles and the labours of life, and furthermore let us admit that in our opinion any attempt to make it too closely resemble the conventicle or the lecture hall would be ill-judged and absurd. Nothing, probably, would be more wearying and insupportable than dramatic entertainments distinctly put forth with an exclusive moral and "improving" object. Assuredly they would not improve upon acquaintance. We had our mysteries and moralities in other days, but even they were relieved by an admixture of lighter matter. "It was a pretty part in the old church plays," says Bishop Harsenet, "when the nimble Vice would skip up nimbly like a Jack-an-Apes into the Devil's necke, and ride the Devil a course, and belabour him with his wooden dagger till he made him roar, whereat the people would laugh to see the Devil so vice-haunted." It was the "licentious pleasantries," as Warton calls them, of these productions, together with the extraordinary and romantic incidents with which they were allied, that rendered *The Fall of Lucifer*, *The Creation*, *The Deluge*, *The Killing of Innocents*, and similar entertainments, so popular with the spectators who witnessed them. But then, as now, dramatic productions written with the special intent of enforcing moral precepts, or of inculcating religious truths, must almost inevitably have failed in that object, from the dulness inherent in all such anomalous and artificial compositions.

We see this in certain works of fiction occasionally published in our own day. Written, not to depict human nature, to portray manners, or to reproduce with vivid colouring the events of an historic past, but simply to illustrate a theory, to propound a dogma, or to solve a problem, of social or political import, the intent out of which they spring obtrudes itself so conspicuously in every page, that the writer stands a very fair chance of ultimately wearying rather than convincing—of losing disciples already made rather than of gaining new converts. It was the great artistic defect of Jerrold as a novelist, that the *purpose* with which he wrote occupied his attention to the exclusion of almost all other considerations, so that the characters of his fictions, and often indeed of his plays, utter the author's thoughts rather than their own.

If the stage is to prosper, then, and fulfil any mission at all, let it eschew dramas written upon a roundhand text, and turn its back upon all productions which are mere sermons in disguise. That the moral should spring out of the play, well and good: but we shall generally have a dreary prospect before us if the play springs out of the moral. Morality cut to pattern is sure to be a misfit.

We may heartily join our voices, therefore, with those who claim the theatre as a place of amusement, and who ask for entertainment within its walls. But this, after all, does not bind us to the whole of their views. For amusement is of various kinds, and according to its nature may be healthy or morbid, elevated or degrading, useful or pernicious. Cock-fighting is an amusement, and when the Whitehall pit basked under kingly favour, it was considered a most fashionable and delightful pastime. Bull-baiting is an amusement, and Paris Garden in its day numbered patrons among the high-born and cultivated, as well as among the plebeian and uninstructed. Rat worrying is also an amusement, and to this day the sporting newspapers will tell us in what direction to bend our steps if we wish to see some vivacious terrier dispatch a certain number of subjects in a certain number of minutes. Pugilistic encounters constitute an amusement, favoured, too, as we saw on a recent occasion, not only by the lower classes, but by the best-bred people in the land, and mentioned with tenderness by the most important organ of public opinion we possess in this country. Amusement has ever been, in fact, and must ever be, of the most diversified nature, as different in its characteristics and organization as in its manifestations and tendencies.

It is impossible, therefore, to place all amusement on the same level, moral or intellectual. Does it make no difference whether we pass an afternoon in gazing around the Salon Carré, or spend it in witnessing a trial of physical prowess between Mr. Sayers and Mr. Heenan? Is the diversion the same whether we obtain it by pulling an oar or by impaling blue-bottles? If one man finds recreation in a visit to South Kensington, and another in sitting over his beer amid the drunken tap-room frequenters of the Dog and Bottle, are both their amusements entitled to take rank in the same category? Is one as commendable as the other, or as worthy of being followed?

Now, what sort of amusement ought we to have in our theatre? Should it be elevating or degrading? Should it refine or brutalize? Should it teach us good manners, or corrupt us with evil counsel? or, lastly, should it exercise a purely neutral influence, and, like the quack doctor's elixir, do us neither harm nor good? Certainly it would be better for the stage to operate thus passively rather than with an actively pernicious effect. But ought we in the interests of art to be satisfied with this poor and pitiful result? If we merely wish to be diverted, let us call in Jack Pudding from the street, pay him a shilling to swallow a string of sausages, or to stand on his head, or to twist his mouth from ear to ear, and surely we may laugh and roar to satiety without stage or theatre at all. But if the drama is an art—and that it is no one will deny—let us cultivate it like any other art, like painting, like sculpture, like music, and strive our utmost to develop it to the utmost. We may not even then reach our ideal; but if we only distantly approach it we shall have made more progress than is possible by simply standing still.

Once admit this principle, that the drama should be cultivated with the loving perseverance and artistic sympathy bestowed upon other arts, and we shall have no difficulty in seeing that it cannot, when thus cultivated, exercise a purely negative influence. It must, by the powerful agencies at its command, be something more than a neutral agent. It must do something more than raise a smile or fill up an hour of leisure. What it should do we need not be long in determining. Jeremy Collier showed himself possessed of no common amount of dramatic insight when he defined the true object and purpose of stage representations—

"The Business of Plays," says he, in his quaint but forcible phraseology, "is to recommend Virtue and discountenance Vice. To show the Uncertainty of Human greatness, the suddain Turns of Fate, and the unhappy Conclusions of Violence and Injustice. 'Tis to expose the singularities of Pride and Fancy, to make Folly and Falsehood contemptible, and to bring everything that is Ill under Infamy and Neglect. This design has been odly pursued by the English Stage. Our Poets write with another View, and are gone into another Interest. 'Tis true were their Intentions fair they might be Serviceable to this Purpose. They have in a great measure the Springs of Thought and Inclination in their power. Show, Musick, Action, and Rhetorick, are moving Entertainments, and rightly employed would be very significant."

Now, the great fault of the drama in our own day is its almost negative character. It certainly does not lay itself open to the censures of Jeremy Collier; but then how far it is from coming up to the requirements of Jeremy Collier! It has no active vices, perhaps, but its passive virtues are almost as reprehensible in these days of moral and intellectual advancement. It is tame, flat, conventional; it has no invention, no ingenuity, no intellectual elevation, no independence, no vital energy. It scarcely takes rank as an art. We are proud of our pictures, we are not ashamed of our sculpture, we hold our general literature in high esteem; but of our drama we make no mention. In a comparatively few metropolitan circles, some interest is still felt in the stage, and here and there the masterly acting of Mr. Webster in *Janet Pride*, the life-like embodiment of Sir Pertinax Macsycophant by Mr. Phelps, or the success of Mr. Robson in his latest character, constitute subjects upon which conversation and colloquial criticism grow animated. But how narrow is the world in which this occurs! The whole of the reading public will have something to say about *Adam Bede*, or *What will he do with It?* but directly you have passed beyond certain metropolitan boundaries, you may inquire in vain after the *Overland Route*, the *Willow Copse*, or *Daddy Hardacre*.

And it is not merely among those who entertain objections to the stage on religious grounds that our theatre is held in such small account. The upper classes, as a body, have almost utterly deserted it. Their amusement they find in the opera—not as some have hastily supposed, because the opera is fashionable, but simply because upon the lyric stage there is a completeness, an affluence of resources, an evidence of managerial taste, and a display of artistic excellence, such as are not to be met with in any establishment where English plays are represented. If Drury-lane could produce *Macbeth* or the *School for Scandal* as Covent-garden can produce the *Prophète* or the *Barbiere*, its programme might be stereotyped for weeks, and not a private box would be obtainable for love or money until the requirements of Belgravia had been fully satisfied.

Nothing indeed can be more pitiable and crestfallen than the stage under its present aspect. It once was full of life and energy, revelling in a powerful influence, which it certainly abused, but which nevertheless showed of what things it was capable. Now the furious beast which Puritanism hunted and struggled with, and which it momentarily overcame, walks abroad unfeared, unheeded, in the public way. The lion has had its claws cut, its teeth have been extracted, even its roar has been tempered to a whisper, and the formidable animal has become as harmless as a lap-dog, and is held in much about the same estimation. The stage has lost all confidence in itself, all courage, all capacity. The broken-down merchant who becomes a messenger in the establishment of which he was once the chief, is not more humble in tone, or more obsequious in manner, than the theatre of the present day. There is scarcely a subject lying out of the beaten dramatic track which it dares to handle. It is afraid to meddle with politics; it shuns all allusion to the great questions which may be agitating the public mind; it shrinks from religion as a poor man shrinks from the elegant and well-furnished church, which he foolishly imagines he is not worthy to enter; it rarely touches upon history, except with timid nervousness; even the manners of the day, the passing follies of the hour, the airy trifles floating in the social atmosphere, and against which the polished shaft of wit and ridicule have ever been directed—even these fail to arouse its slumbering energies. It goes on at a jog-trot pace, the embodiment of a commonplace respectability, which, in its eagerness to offend no susceptibilities, to awaken no antagonism, to pass beyond no established formula of thought and speech, becomes pre-eminently tame, servile, humdrum, harmless, and contemptible.

True, the stage, as though still in its teens, has a guardian, a strange anomalous protector, called a censor, whose mission it is to look after the little boy, and see that he does not use any bad language, throw stones, get into evil company, or read Tom Paine; and this fact, doubtless, accounts to some extent for the timidity and tameness to which we have made allusion. But how is it such an absurd officer is allowed to exist a day in free England? Why! if the stage lad had any spirit, he would turn round upon the protector by whom he is followed, hit out at that well-dressed inculcator of propriety, and serve the official Mentor exactly as Tom Brown served Slogger Williams. After a little sparring, and very few rounds, we should hear no more of the dramatic censor, depend upon it.

Fully nine-tenths of the works now brought out upon the English stage are utterly colourless and conventional. They reproduce conventional characters, they employ conventional phraseology, they embody conventional ideas, they depend for the interest they excite upon conventional incidents, developed through the agency of conventional plots. Time advances, but the stage stands still, and in this way it happens that the pictures of life represented at the theatre apply neither to the past nor to the present, nor, indeed, to any day. To paraphrase the eulogy bestowed upon Shakespeare's masterpieces, they are not of an age, but of no time.

Taking all these circumstances into account, it is strange to find many people of by no means illiberal or narrow-minded views, still shunning the theatre on principle ; shunning it in no bigoted, sectarian spirit, but on the broad ground of morality and Christian decorum ; believing it the same pest-house of iniquity it is held to be in Puritan traditions ; regarding it as Satan's own playground, and feeling quite ready to disinherit son or daughter of theirs, who should enter into its unhallowed precincts. Now, we have no wish to treat such views with levity, or to throw any ridicule upon convictions which we know are entertained in full sincerity. Still we cannot help expressing the opinion that those views and convictions rightly belong to another period, and are not justified by the present condition of our stage.

Doubtless, there is to many minds a fascination in the theatre, which, if unduly yielded to, would lead to no good result. But may not the same be said of all amusements, even those which we find ministers of religion openly and zealously advocating in the present day ? Cricket, for instance, is a very exhilarating, beneficial, and manly pastime, but if Master Bob or young Mr. William were to give up all their time to it, neglect school studies for it, and plainly show that their whole thoughts were absorbed in the game, their father would assuredly be justified in pulling up the wickets, burning the bats, and pitching the balls into a horse-pond. Again, there is a fascination in the society of women, more especially perhaps if they be young, pretty, intelligent, accomplished, well-bred, amiable, and free from affectation. But are we therefore to set it up as a rule of our lives, that we must never enter the presence of these syrens, lest, once drawn across the charmed circle, we find ourselves without power to return ? The theatre, as an amusement, must of course be followed like any other, with reason and in moderation, and when thus followed, its results will assuredly not be of a kind to excite alarm.

But do the opponents of the stage really know what sort of an amusement it is which they hold in such deep abhorrence ? Let them enter with us just for once into a good metropolitan theatre. If they don't like the approaches to Wych-street, we will pass by the Olympic, and go up the Strand to the Adelphi ; and when at that house, we can easily run along to the Haymarket, or take a cab to the distant Princess's. It is immaterial which, so we will enter the first we reach.

We look around, and see an audience as decorous and well behaved as that which assembles in Exeter Hall. Hush ! Even as we enter they call upon us not to break in upon the earnest attention with which they are listening to what is passing upon the stage. Our companions are evidently impressed, but not yet quite at ease. They gaze in every direction, sweeping boxes, pit, and gallery alike with looks of mistrust and suspicion. Don't be alarmed, gentlemen, the painted syren you expected to see is not here; she has long since received her *congé*, long since has ceased to have the run of the house. If she wants to ensnare youth and innocence, she has no greater opportunities afforded her here, than in your own churches and chapels. If she be here at all, it is only by conforming to the same rules of decorum and good behaviour, under favour of which she would at any time obtain a free seat in your temples, or if nicely and unobtrusively dressed, be ushered into a well hassocked pew. Cease your scrutiny, therefore, gentlemen, and note what is passing upon the stage.

You heard that burst of satisfaction, followed by that long and hearty round of applause. You wonder what occasioned it. Some witticism in favour of profligate habits, or some ridicule bestowed upon virtuous manners, you doubtless imagine. Not at all. That poor man upon the stage, who you can see is in terrible misery has been offered a large sum by a rich banker, not a member of the bloated aristocracy, if he will perjure himself. The tempted has but replied to the tempter: "No, sir, the pangs of poverty are hard to bear, but the wealth purchased by crime would bring with it even deeper agony;" and the audience have expressed their approval with that extravagance of gratification which wakes up so many slumbering echoes. A very scandalous and vice-loving audience, it is evident.

Another burst of applause. Surely that must have been occasioned by some objectionable incident, or some improper speech. No; the heroine hears the character of her father falsely stigmatised, and is defending it with filial zeal. "'Tis false. I am his child," are all the words she has uttered, and yet the pit is shedding tears of sympathy, and in the gallery there is not a dry eye, or a pocket handkerchief free from dampness.

And now another manifestation of delight is expressed by peals upon peals of laughter. This time something ribald or profane must assuredly have been uttered, or the entire audience would not thus give vent to its mirth. Once more, No! The comic man has merely knocked down a free-and-easy fine gentleman, who was persecuting an innocent young maid with his amorous impertinence, and the spectators whom you thought utterly won over to the wrong cause, and devoid of all sympathy with aught save evil cannot contain themselves for joy. Have you seen enough, gentlemen, or will you stay longer? If you do, you will witness nothing but entertainments of much the same character; and though in time you may get somewhat weary of them, as we are, you will scarcely be able to charge them with any graver blemish than their monotony and prosiness.

Whatever may be the faults of the stage, immorality cannot be said to form one of them. Run over the lists of pieces produced in London during the last ten years. Here and there we shall find one containing incidents which betray too decidedly their French origin; but, as a general rule, the works are utterly free from noxious matter. It is the first occupation, indeed, of the English playwright in adapting the dramas of France to our own stage, to expunge from them all objectionable passages. He knows that an English audience has no sympathy, for instance, with adultery, under whatever cover of sentimentality it may be introduced. Out, therefore, goes the luckless hero or heroine, whose engaging frailties have seduced away the tears of the Ambigu or the Vaudeville. By the time hero or heroine appear before British audiences, the driven snow is not fairer than their innocence and purity. When the stage offends against any of the moral laws, it would seem to do so from mere poverty of invention and dulness of judgment—not from any love of viciousness, or any desire to corrupt. Its greatest sins are blunders, and these, it is only justice to say, are committed but seldom.

Here, perhaps, we shall be met by the question, If the stage is thus pure and innocent, what more can you ask of it? Jeremy Collier merely expects it to recommend virtue, and discountenance vice; are you to be more exacting than he? And if it already fulfils these conditions, how can it be looked upon as falling short of the requirements of its religious critic? Our answer to these objections is soon expressed. The purity and innocence of the drama we look upon as simply negative merits. We expect these qualities, as a matter of course, in the present day—as a result, indeed, of the general tone of society, and the prevailing healthiness of our literature. When our novelists and poets are distinguished by the delicacy and elevation of their sentiments, it would be strange, indeed, if the stage were so far behind the age as to be corrupt and vicious, intent only upon contaminating the public mind. We claim from it, therefore, decency of expression and soundness of purpose, as something we have an absolute right to look for.

But have we not a right to look for something more? Are we satisfied with a novel simply because it is full of excellent sentiments and impregnated with the most amiable piety? Does a poem take up the post of honour upon our bookshelves because the author despises meanness, has a horror of guilt, and expresses these laudable feelings in metrical slipshod? Does the essay, or even the sermon, pass muster, unless it contains something more edifying than common-place views clumsily expressed and badly arranged? Assuredly not. In the novel, the poem, the essay, even in the sermon, we look for literary merit, for originality, for force, for energy, for all the various qualities indeed which go to make up excellence in a work of art. And why should we not look for the same in a tragedy, comedy, farce, burlesque, or domestic drama?

This, then, is our complaint against the stage—not that it is immoral, but that it is tame, commonplace, and conventional. The sermon is perfectly sound in doctrine, it inculcates nothing but excellent precepts, it contains not an opinion from which we can dissent, and yet its effect is sedative and unsatisfactory. The similes limp with age, the sentences move awkwardly, the arguments totter under the ever-increasing burden of words they are compelled to bear. Not a bad sort of sermon, if listened to for the first time, but coming rather flatly after being heard with a few variations every Sunday from January to December.

It may fairly be said that every department of literature shows signs of intellectual activity and of vital force, except that of the stage. In history, biography, political economy, philosophy, theology, the sciences, we have writers who labour with rare earnestness and perseverance, and who bring to their work highly-cultivated minds, besides talents of the first order. In fiction, we have a host of authors whose works occupy no insignificant position in our literature—Mrs. Gaskell, Miss Muloch, George Eliot, Charles Reade, Kingsley, Antony Trollope, Wilkie Collins, Lever, besides the three great masters of their art—Bulwer, Dickens, and Thackeray. But on the stage there is no mental activity, no signs of intellectual life. A few years ago we might have claimed Jerrold, Bulwer, Knowles, Talfourd, Lovell, Marston, as writers who reflected honour upon our theatre; now the list must dwindle down into a single name, and in Tom Taylor we are compelled to acknowledge we have almost the only distinguished representative of our dramatic literature. *Totus mundus agit histrionem*, says Petronius, but where are we to find the dramatists?

In the preceding remarks, we have spoken of the drama with special reference to the works represented in what are called the West-end theatres, and which taken collectively may fairly be said to constitute the English stage. But we should leave the subject incompletely developed, did we not allude to another class of dramatic productions which form the staple amusement at another class of theatres. For in these latter works we meet with a fresh subject, the literature, such as it is, of the minor stage.

When Charles Mathews published his witty but somewhat flippant pamphlet upon the copyright treaty between France and England, he gave in it a vivid description of one of our minor theatres.

"The Victoria," says he, in his rattling, farcical manner, "is a model house, the type of a school to which it gives its name. It is the incarnation of the English domestic drama, or rather of the drama of English domestics. There you will always find the truest picture of virtue in rags and vice in fine linen. There flourish the choicest specimens of all the crimes which make life hideous—robbery, rape, murder, suicide. It is a country abounding in grand combats of four—a region peopled with angelic maid-servants, comic housebreakers, heroic sailors, tyrannical masters, poetical clodhoppers, and diabolical barons. The lower orders rush there in mobs, and in shirt sleeves, applaud frantically, drink ginger beer, munch apples, crack nuts, call the actors by their Christian names, and throw them orange-peel and apples by way of bouquets." Allowing for a little satirical colouring, this is on the whole a by no means overcharged picture, and with a few unimportant modifications, it might be accepted as applying to the whole of our minor theatres. But there is one point in the description upon which special emphasis must be laid. It is the fondness of the minor stage for evil deeds. Not that crime is applauded, or held up as a pattern to be imitated. On the contrary, it is, as a rule, held up to detestation and loathing. In the secondary theatres of the metropolis, as in the theatres of superior rank, virtue generally meets with its due re ward, and vice receives condign punishment. Pieces like *Jack Sheppard*, in which the hero is a sentimental rogue, carrying with him the sympathies of the audience, may fairly be set down as exceptional. In almost every instance, the really bad characters are painted in the deepest black, and the really good appear under an aspect of moral excellence utterly spotless in its purity. Thus it becomes almost impossible for the sympathies of the audience to flow in a wrong direction when they flow at all. The virtuous peasant and servant girl proclaim their virtue in every speech they utter, and in every action of their dramatic lives; while the usurping baron and unscrupulous assassin never open their lips without acknowledging their rascality, and letting the spectators into the secret of all their evil deeds.

This, to a great extent, may be considered as the saving merit of the minor drama. If it sentimentalized immorality, represented crime under an æsthetical aspect, and appealed thus to the morbid rather than to the healthy sympathies of its patrons, the effect could not fail to be highly pernicious. In France, where unwholesome popular literature is the rule instead of the exception, it is impossible not to believe that novelists and playwrights count for something among the causes which lead to the diseased tone of social sentiment, and ultimately to the development of crime. We find, at all events, that those offences which indicate strong morbid tendencies on the part of the perpetrators, flourish to a much greater extent among our neighbours than among ourselves; and we know that those tendencies must be encouraged and strengthened by the novels and plays most generally admired. Some serious crimes are, indeed, really common in France, while in England they are compartively rare. Parricide and infanticide, for instance, occupy a very prominent place upon the French criminal records, while upon our own they are completely in the background. Serious crimes, too, against the person—such as murder and attempts to murder—are committed in France, to fully twice the extent they are committed in England. The same results are exhibited in another and not less terrible class of offences, viz., rape and attempts at rape. We do not put forward these statistics in any dogmatic spirit of inference, or attach too much importance to the support they may appear to give to our views. Still the contrast supplied by a comparison of the criminal balance-sheets of the two countries is at least worthy of mention here, and its value is not diminished when we find that it is only in the minor crimes—such as petty thefts, which do not carry with them evidence of highly morbid tendencies, but rather of want and hunger, or laziness—that England is conspicuously in excess of France.

To say truth, the minor stage is too ignorant, stupid, bungling, and unimaginative to do much harm. It long ago came to the conclusion that its patrons delighted in bloodshed, and it has done nothing but act upon that conclusion ever since. But it is only in the hands of genius that a single theme can be manipulated for any length of time without taking awkward and abnormal shapes. At first, there was no doubt a terrible truthfulness and startling literality in the more painful and exciting incidents of the minor stage, but ordinary minds could not for ever arrange new combinations of horror, or always draw from the deep well of crime without exhausting the supply at the bottom. Murder, suicide, burglary, abduction, were so frequently made use of, that they grew at last threadbare subjects. Even when patched up with a supernatural lining they were found scarcely fit for service. From bad to worse was thenceforth a natural transition. When horror of ordinary quality lost its influence, horror above proof was resorted to. The usual effect of exaggeration has followed. In the present day the melo-dramatic horrors of the minor stage are merely melo-dramatic absurdities. They scarcely interest the most uneducated audience, while to the educated they are simply ridiculous. No greater diversion can be imagined for those who love a hearty laugh than is offered by a genuine Victoria or Surrey melo-drama, for both productions are on about the same intellectual footing, when represented by a company accustomed to the work. The whole piece is a mass of sanguinary puerility. The characters are not human beings, they are mere stage abstractions. They do not talk or declaim, they rant and bawl. They come and go, they laugh and scowl, they bless and anathematize, they poison and stab, they shoot and garotte, they die and live; but whatever they do carries with it a sense of falseness, of unreality, of right down absurdity. The whole drama is a jumble, incoherent and meaningless. There is no plan anywhere visible. Murders are committed, virtuous deeds are performed, but they have no motive, no significance—all is pell-mell or chaos. The drama of the minor stage is, in fine, mere bloodthirsty balderdash, with scarce a glimmering of real wit, humour, pathos, or invention, and, as an intellectual emanation, would almost reflect disgrace even upon a metropolis of Hottentots or Feejee Islanders—to say nothing of civilized Englishmen.

Mr. Charles Reade, in the curious work he has lately published under the title of *The Eighth Commandment*, indicates what he regards as the cause of our theatrical degradation, and points out the remedy. According to this writer we have ceased to have a drama worthy of the name, simply because we have ceased to pay for it. What we used once to buy we now steal, and the French stage is the affluent storehouse in which we commit our depredations. The English dramatist has thus been driven out of the field, and the English dramatic pirate or vampire hack has taken his place. No man of education and ability can now afford to write for the theatre, his productions being at once placed in unfair competition with those which have cost a merely nominal amount of time and mental labour. The whole question is thus one of money. Offer a fair pecuniary inducement to good authors, and the stage will soon give evidence of the intellectual vitality observable in other departments of literature. To show into what a state of decay and discredit the art of the dramatist has fallen, Mr. Reade gives a few figures of convincing significance, and the accuracy of which cannot be questioned. Thus a recent trial disclosed to us that the average price of a new play in many flourishing London theatres is 4*l.*, but we also hear of five pieces being sold for 3*l.*, and of three separate lists of plays being offered to a manager for thirty shillings and twenty shillings per annum. These, of course, were works by mere hacks, and were intended only for the minor stage. But if we rise in the scale of merit, the rate of remuneration does not mount to a very high level. *Black-eyed Susan*, for instance, brought its author only 60*l.*, though the actor who played the leading character realized 4000*l.* by the impersonation; for *Masks and Faces*, Messrs. Tom Taylor and Charles Reade received 150*l.*, and for *Two Loves and a Life*, 100*l.*; while for the most successful play of modern times, *The Lady of Lyons*, only 500*l.* was paid.

In contrast with these figures may be placed the prices paid to English dramatists in another generation of playgoers. Thus, for Dr. Johnson's dull play of *Irene*, 300*l*. was given; for *The Good-natured Man*, 500*l*.; for *She Stoops to Conquer*, 800*l*.; for Holcroft's *Follies of a Day*, 600*l*., and 300*l*. for the copyright; for *The Road to Ruin*, 900*l*., with 400*l*. additional for the copyright; for Colman's *John Bull*, 1000*l*.; for *The Brothers*, by Dr. Young, 1000*l*.; and for *Marianne*, by Elijah Fenton, 1000*l*. We need not adduce further evidence upon this point. Enough, we think, has been brought forward to show that, if properly cultivated, the stage might become a powerful engine of mental culture, though evidently it must begin by growing honest if it would exert all the influence of which it is capable.

---

